# Intra-Articular Steroid Injection with or Without Pulsed Radiofrequency in Knee Osteoarthritis: A Retrospective Comparative Study

**DOI:** 10.3390/medicina62081492

**Published:** 2026-08-03

**Authors:** Emel Basar, Nevcihan Sahutoglu Bal, Gulcin Babaoglu, Hatice Babaoglan, Erkan Yavuz Akcaboy

**Affiliations:** Department of Pain Medicine, Ankara Bilkent City Hospital, Ankara 06800, Türkiye; nevci_sahut@hotmail.com (N.S.B.); gulcinpektasli@gmail.com (G.B.); haticebabaoglan@hotmail.com (H.B.); akcaboyyavuz@gmail.com (E.Y.A.)

**Keywords:** knee osteoarthritis, intra-articular corticosteroid injection, pulsed radiofrequency, visual analog scale, WOMAC, treatment outcome

## Abstract

*Background and Objectives*: Knee osteoarthritis is a major cause of chronic pain and disability. Because the benefit of intra-articular corticosteroid injection is often limited, this study compared fluoroscopy-guided corticosteroid injection alone with corticosteroid injection combined with pulsed radiofrequency (PRF). *Materials and Methods*: This retrospective comparative study included 386 consecutive patients with symptomatic knee osteoarthritis treated with fluoroscopy-guided intra-articular corticosteroid injection alone (IAS, *n* = 190) or combined with PRF (IAS + PRF, *n* = 196). Pain and function were assessed using the Visual Analog Scale (VAS) and Western Ontario and McMaster Universities Osteoarthritis Index (WOMAC) at baseline and 1 week, 1, 3, and 6 months. The primary endpoint was the 6-month VAS score. Baseline-adjusted linear mixed-effects models were the primary analyses, with propensity score-matched analyses as sensitivity analyses. *Results:* Both groups showed significant improvements in pain over time. In the primary baseline-adjusted analyses, adding PRF was associated with greater early- and mid-term pain reduction, including significantly lower VAS scores at the 6-month primary endpoint and higher rates of clinically meaningful pain improvement (≥50% response). However, this adjusted difference was modest (below the minimal clinically important difference) and was no longer statistically significant after propensity score matching (6-month VAS median 5.0 in both groups; *p* = 0.763), indicating that baseline imbalance contributed to the apparent advantage. *Conclusions:* In baseline-adjusted analyses, adding PRF to intra-articular corticosteroid injection was associated with more favourable early- and mid-term pain outcomes than corticosteroid injection alone, although the difference was modest and was not confirmed after propensity score matching. Given the retrospective, non-randomized design and baseline imbalance, these findings should be considered hypothesis-generating. Prospective randomized, sham-controlled studies with longer follow-up are needed to clarify the role of PRF as an adjunctive treatment for knee osteoarthritis.

## 1. Introduction

Osteoarthritis (OA) is the most common chronic degenerative disease of synovial joints and is increasingly recognised as a disorder affecting the entire joint rather than articular cartilage alone. OA is characterised by progressive disruption of the structural and biomechanical integrity of articular cartilage, accompanied by low-grade synovial inflammation, subchondral bone remodelling, and osteophyte formation, ultimately leading to chronic pain, functional impairment, and reduced quality of life [1]. In 2020, OA affected an estimated 595 million people worldwide, and its global burden is projected to increase by approximately 75% by 2050, underscoring the growing need for effective and durable treatment strategies [2].

Articular cartilage is a highly specialised avascular connective tissue with a very limited intrinsic capacity for regeneration, making the preservation of its structural and functional integrity essential for normal joint function [3,4]. Because cartilage lacks a direct vascular supply, chondrocytes depend primarily on diffusion of nutrients and metabolites from the synovial fluid, while physiological mechanical loading facilitates nutrient transport and helps maintain extracellular matrix homeostasis [5,6,7,8,9]. The highly organised extracellular matrix, composed predominantly of type II collagen, proteoglycans, and water, provides the tensile strength, viscoelasticity, and compressive resilience required for normal joint biomechanics [3,10,11,12]. Progressive disruption of these structural and biomechanical properties impairs cartilage function, accelerates extracellular matrix degradation, and contributes to the onset and progression of OA [13,14].

As OA progresses, extracellular matrix homeostasis becomes increasingly disrupted, resulting in proteoglycan depletion, disorganisation of the type II collagen network, and deterioration of the mechanical properties that are essential for normal cartilage function [14,15,16,17]. These structural changes are accompanied by abnormal chondrocyte activation, increased production of matrix-degrading enzymes, and persistent low-grade synovial inflammation, which collectively accelerate cartilage degeneration [15]. In addition, alterations in the synovial environment impair nutrient transport and compromise cartilage homeostasis, further limiting the already poor regenerative capacity of articular cartilage and contributing to disease progression and functional decline [18,19].

Recent biomechanical evidence further demonstrates that osteoarthritis is characterized by progressive deterioration of the extracellular cartilage matrix, particularly the depletion of type II collagen and proteoglycans, resulting in impaired load distribution, reduced viscoelasticity, increased joint friction, and progressive exposure of the subchondral bone. These structural and biomechanical alterations contribute directly to pain, functional impairment, and disease progression, highlighting the need for multimodal treatment strategies that address both structural degeneration and symptom control [20].

Beyond biomechanical deterioration, molecular and cellular mechanisms also contribute to OA progression; in particular, dysregulation of trace-element homeostasis—for example, altered iron, copper, zinc, and selenium levels—has been associated with oxidative stress, chondrocyte dysfunction, extracellular-matrix degradation, and synovial inflammation, further accelerating cartilage degeneration [21]. These observations reinforce the need for treatment strategies that address both the nociceptive and the biological components of knee osteoarthritis.

Current management of OA remains primarily focused on symptom relief and preservation of physical function, as no currently available treatment has consistently demonstrated disease-modifying efficacy. Conservative treatment options include patient education, weight management, structured exercise and physical rehabilitation, pharmacological therapy, intra-articular injections such as corticosteroids, hyaluronic acid and platelet-rich plasma, as well as interventional procedures including genicular nerve ablation. Although these approaches may provide meaningful clinical improvement, their therapeutic effects are frequently temporary, and none has consistently demonstrated the ability to halt or reverse the progressive structural degeneration of the joint. For patients with persistent symptoms and advanced disease, total knee arthroplasty remains an effective treatment option; however, many individuals are either poor surgical candidates because of advanced age or multiple comorbidities or prefer to delay surgery. Consequently, there is an increasing need for safe, minimally invasive treatment strategies capable of providing sustained pain relief and functional improvement [22].

Conventional thermal radiofrequency ablation (RFA) is supported by high-level evidence for the management of several chronic pain syndromes [23]; however, its use around synovial joints may be constrained by risks such as motor fiber injury, neuritis, and anesthesia dolorosa due to irreversible neural destruction [24].

Among these emerging minimally invasive approaches, pulsed radiofrequency (PRF) has emerged as a promising neuromodulatory intervention owing to its ability to modulate pain signalling without producing irreversible neural injury. Unlike conventional thermal radiofrequency ablation, PRF delivers short bursts of high-voltage current while maintaining tissue temperatures below 45 °C, thereby generating a strong electric field without inducing thermal neurodestruction. Experimental studies suggest that this electric field may suppress pro-inflammatory cytokine release, inhibit microglial activation, induce long-term synaptic depression, and promote chondrocyte proliferation and extracellular matrix synthesis, providing a biological rationale for its application in degenerative joint disorders [25,26,27,28,29].

Early clinical studies have reported encouraging improvements in pain and function following intra-articular PRF in patients with knee OA. Karaman et al. reported favourable outcomes in a cohort of 31 patients with knee OA treated with intra-articular PRF [30], and some evidence suggests that PRF may outperform intra-articular corticosteroid injections in selected populations.

Although preliminary clinical studies have reported encouraging improvements in pain and function following intra-articular PRF in patients with knee OA, the available evidence remains limited, particularly regarding its use in combination with intra-articular corticosteroid injection and with respect to medium-term pain outcomes. Therefore, the present retrospective comparative study was undertaken to compare pain outcomes following intra-articular corticosteroid injection alone and corticosteroid injection combined with PRF in patients with knee OA refractory to conservative treatment.

## 2. Materials and Methods

### 2.1. Study Design and Ethics

This retrospective comparative study was approved by the Institutional Ethics Committee of our institution (approval no. E1-24-736, 20 November 2024). Medical records and follow-up documentation of patients with knee osteoarthritis who presented to a tertiary referral pain medicine centre between 1 January 2022 and 31 December 2024 were reviewed. The study was conducted in accordance with the ethical standards of the Declaration of Helsinki. A completed STROBE checklist is provided as Supplementary Table S1.

### 2.2. Participants

Inclusion criteria were age ≥ 18 years; clinically and radiologically confirmed knee osteoarthritis (Kellgren–Lawrence grade II–IV) [31]; persistent knee pain for >3 months; and inadequate response to conservative treatment, including nonsteroidal anti-inflammatory drugs (NSAIDs), intra-articular hyaluronic acid, and physiotherapy. Exclusion criteria were local or systemic infection; coagulopathy or current anticoagulant therapy; known allergy to corticosteroids or contrast agents; knee surgery within the preceding 12 months; intra-articular injection (corticosteroid, hyaluronic acid, or platelet-rich plasma) within the previous 6 months; inflammatory arthropathies (e.g., rheumatoid arthritis, psoriatic arthritis, or gout); serious systemic comorbidities that could interfere with treatment or follow-up; pregnancy; mental disorders affecting adherence to follow-up; and incomplete follow-up or missing outcome data. Patients with prior intra-articular hyaluronic acid treatment were eligible only if the injection had been administered at least 6 months before enrolment. In patients with bilateral knee osteoarthritis, only the more symptomatic knee was designated as the index knee and included in the analysis. Patients who underwent simultaneous bilateral procedures were excluded to ensure that each patient contributed only one knee to the statistical analysis.

### 2.3. Group Allocation

This was a non-randomised, retrospective study. The decision to perform IAS + PRF rather than IAS alone was made by the treating fellowship-trained interventional pain specialist on the basis of individual clinical judgement at the time of the procedure and the intra-procedural technical suitability and availability of pulsed radiofrequency equipment. No predefined patient-level algorithm governed the choice of procedure. Because allocation was determined by clinical and technical considerations rather than by randomisation or a fixed protocol, the potential for selection bias is explicitly acknowledged as a key limitation of this study and is addressed analytically through covariate-adjusted modelling and propensity score matching (Section 2.6).

**Figure 1:**
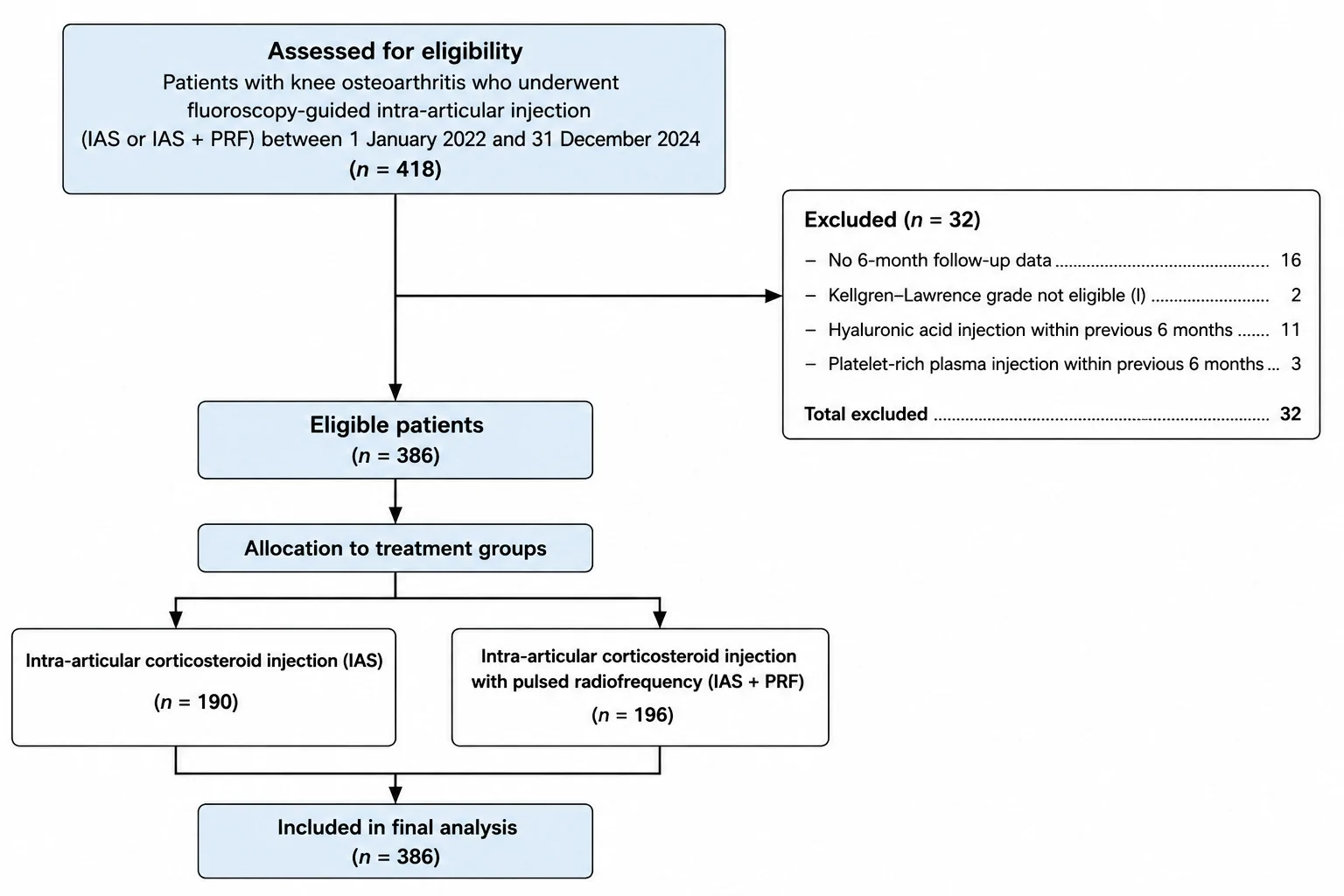
Flow diagram of patient selection. Abbreviations: IAS, intra-articular corticosteroid injection; PRF, pulsed radiofrequency.

A total of 386 consecutive eligible patients with knee osteoarthritis who underwent fluoroscopy-guided intra-articular steroid injection (IAS) or intra-articular steroid injection combined with pulsed radiofrequency (IAS + PRF) during the study period were included in the final analysis and categorised into the IAS group (*n* = 190) and the IAS + PRF group (*n* = 196). A patient flow diagram is presented in Figure 1. All procedures were performed at a single centre by fellowship-trained interventional pain specialists using a standardised institutional protocol.

### 2.4. Outcome Measures

The primary endpoint was the change in pain intensity from baseline, assessed using the Visual Analog Scale (VAS) at 6 months. Secondary endpoints included VAS scores at 1 week, 1 month, and 3 months; the proportion of patients achieving the minimum clinically important difference (MCID) in VAS; and the proportion of patients classified as responders based on ≥30% or ≥50% reductions in VAS from baseline.

Pain intensity was assessed using the 10-point VAS (0 = no pain, 10 = worst imaginable pain). Baseline functional status was assessed using the WOMAC, version 3.1, total score (range 0–96; higher scores indicate greater disability). Reliable prospectively recorded WOMAC follow-up values were not available in the source records; WOMAC was therefore used only to characterise baseline functional status and to inform covariate adjustment and propensity score matching, and longitudinal functional outcomes were not analysed. VAS was recorded at baseline and at 1 week, 1 month, 3 months, and 6 months and was the sole longitudinal outcome.

### 2.5. Procedures

For all procedures, patients were positioned supine with the knee slightly flexed over a support. Standard non-invasive monitoring, including blood pressure, pulse oximetry, and electrocardiography, was applied. Following aseptic skin preparation with 10% povidone-iodine, local anaesthesia was administered using 2% prilocaine (Pricain^®^ 2%, 20 mL; Polifarma, Tekirdag, Turkey). An antero-posterior fluoroscopic image of the knee joint was obtained with C-arm fluoroscopy. Under fluoroscopic guidance, a 22-gauge Quincke spinal needle was advanced into the intra-articular space via an anterolateral approach, and correct needle placement was confirmed using radiopaque contrast injection.

In the IAS group, a mixture of 40 mg methylprednisolone acetate (Depo-Medrol^®^, 2 mL; Pfizer, Istanbul, Turkey) and 2 mL of 2% lidocaine was injected into the joint space. In the IAS + PRF group, a unipolar 22-gauge radiofrequency cannula (10 cm length, 5 mm active tip) was positioned intra-articularly under fluoroscopy, and its placement was verified with contrast. Sensory stimulation at 50 Hz and motor stimulation at 2 Hz (1 V) were performed to confirm the absence of neural activation. PRF was applied at 42 °C for three cycles of 120 s using standard parameters (45 V, 2 Hz pulse frequency, 1 ms pulse width) with a NeuroTherm NT 1100 generator (Abbott Medical, Abbott Park, IL, USA). No pharmacological agents were administered during PRF. Following PRF, the same steroid–local anaesthetic mixture used in the IAS group was injected into the joint space, ensuring that the only procedural difference between groups was the application of pulsed radiofrequency energy. All adverse events and complications were documented during follow-up visits.

### 2.6. Statistical Analysis

Statistical analyses were performed using Python (version 3.11) within a Jupyter environment, with the pandas (v2.0.3), statsmodels (v0.14.0), scipy, and scikit-learn libraries. Descriptive statistics are presented as mean ± standard deviation (SD) for normally distributed continuous variables and as median (interquartile range, IQR) for non-normally distributed variables. Categorical variables are reported as frequencies and percentages. The normality of continuous variables was assessed using the Shapiro–Wilk test and visual inspection of Q–Q plots. Baseline characteristics were compared using Student’s independent-samples *t*-test (parametric data), the Mann–Whitney U test (non-parametric data), and Pearson’s chi-square test (categorical variables).

Primary analysis: The primary analytical approach consisted of linear mixed-effects models (LMM) with individual patient random intercepts. Baseline VAS was incorporated as a covariate in the primary model, alongside treatment group, categorical time (follow-up visit), their interaction (Group × Time), and the following pre-specified confounders: age, sex, body mass index (BMI), and Kellgren–Lawrence grade. This baseline-adjusted parameterisation was pre-specified prior to outcome analysis to account for observed between-group differences in baseline characteristics. Sensitivity analyses using continuous time (weeks) were performed to assess the linearity of the treatment trajectory.

Clinical significance: Patients were classified as VAS responders if they achieved ≥ 30% or ≥50% reduction from baseline VAS at each time point. The MCID for VAS was defined as ≥ 1.5 points of absolute reduction from baseline, consistent with established thresholds in knee osteoarthritis [32,33]. Between-group responder and MCID rates were compared using Pearson’s chi-square test. Proportions are reported with Wilson score 95% confidence intervals (CI); between-group differences in proportions with Newcombe hybrid-score 95% CI.

Propensity score matching (PSM): To further address baseline heterogeneity, a sensitivity analysis using 1:1 nearest-neighbour propensity score matching was conducted. Propensity scores were estimated by logistic regression using six covariates: age, sex, BMI, Kellgren–Lawrence grade, baseline VAS, and baseline WOMAC. Matching used a calliper of 0.2 SD of the propensity score. Balance was evaluated using the standardised mean difference (SMD), with values < 0.10 conventionally considered indicative of adequate balance; 95% CIs for SMD estimates were obtained via bootstrap resampling (2000 iterations, percentile method) to quantify estimation uncertainty given the matched sample size.

Cross-sectional comparisons between groups at each time point, and absolute (Δ) and percentage (%) changes from baseline computed at the patient level, were treated as exploratory and compared using the Mann–Whitney U test. All tests were two-tailed; statistical significance was set at *p* < 0.05. No formal a priori sample-size calculation was performed; all eligible consecutive patients treated during the study period were included. Apart from the pre-specified primary endpoint (6-month VAS), analyses across the multiple follow-up time points, responder thresholds, and Kellgren–Lawrence strata were exploratory and were not adjusted for multiple comparisons, and the corresponding *p*-values should be interpreted accordingly.

## 3. Results

### 3.1. Baseline Characteristics

A total of 386 patients were included: IAS group (*n* = 190) and IAS + PRF group (*n* = 196). Baseline demographic and clinical characteristics are summarised in Table 1. The groups were similar in age (69.71 ± 10.62 vs. 69.76 ± 9.43 years; *p* = 0.965), BMI (31.62 ± 2.92 vs. 31.75 ± 2.70 kg/m^2^; *p* = 0.636), and sex (female 70.5% vs. 70.4%; *p* = 1.000), but three baseline variables differed significantly: baseline VAS was marginally higher in the IAS + PRF group (median 8.0, IQR 8.0–8.0 vs. 8.0, IQR 7.0–8.0; *p* = 0.040); the Kellgren–Lawrence grade distribution differed (*p* = 0.015; Table 2); and baseline WOMAC was substantially higher in the IAS group (median 65.0, IQR 45.0–80.0 vs. 50.0, IQR 35.5–70.0; *p* < 0.001), indicating greater baseline functional impairment. These imbalances were addressed in the primary analysis through covariate adjustment and in the sensitivity analysis through propensity score matching.

### 3.2. Cross-Sectional Comparisons of VAS

**Figure 2:**
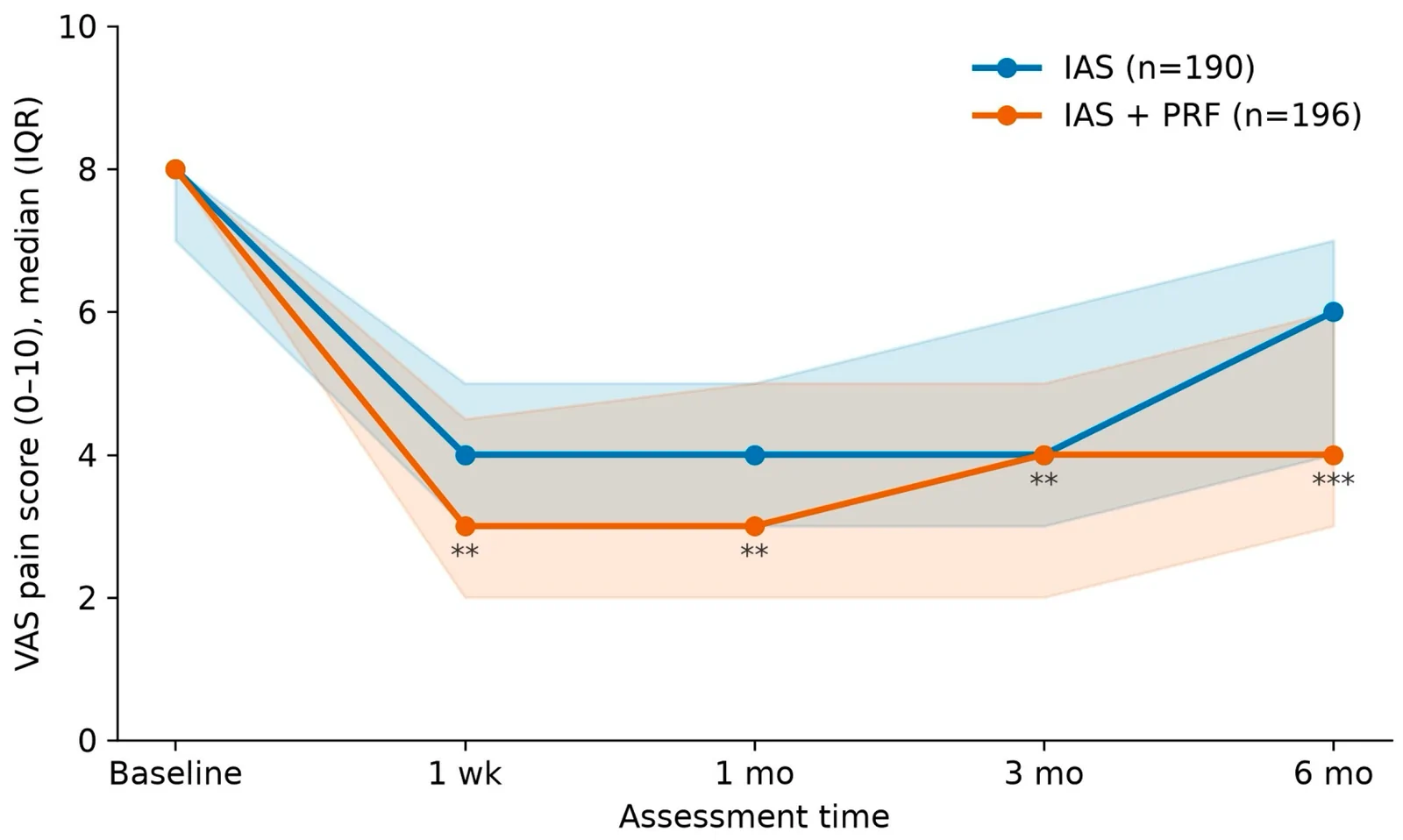
Median VAS pain trajectory by treatment group (unadjusted). Lines: group median; bands: interquartile range. Between-group Mann–Whitney U tests: ** *p* < 0.01, *** *p* < 0.001. IAS *n* = 190, IAS + PRF *n* = 196.

Median VAS scores at each follow-up time point are presented in Table 3 and Figure 2. The IAS + PRF group showed consistently lower VAS scores at all follow-up visits: 1 week (3.0 vs. 4.0; *p* = 0.002), 1 month (3.0 vs. 4.0; *p* = 0.004), 3 months (4.0 vs. 4.0; *p* = 0.003), and 6 months (4.0 vs. 6.0; *p* < 0.001), despite marginally higher baseline VAS. These cross-sectional comparisons are exploratory; primary inference rests on the baseline-adjusted models (Section 3.4).

### 3.3. Temporal Changes: Absolute (Δ) and Percentage (%) Differences

Absolute and percentage changes from baseline are presented in Table 4. For VAS, both absolute and percentage reductions were significantly greater in the IAS + PRF group at all time points (all *p* < 0.001). At 6 months, median VAS reduction was 4.0 points (IQR 2.0–5.0) in the IAS + PRF group versus 2.0 points (IQR 1.0–4.0) in the IAS group; median percentage reductions were −50.0% and −28.6%, respectively.

### 3.4. Primary Analysis: Linear Mixed-Effects Model (VAS)

#### VAS—Baseline-Adjusted LMM

Baseline-adjusted LMM analysis demonstrated significant VAS reductions over time in both groups (all time points vs. baseline, *p* < 0.001). The main effect of treatment group was significant (β = −0.718, *p* < 0.001), indicating lower average VAS in the IAS + PRF group after adjustment for baseline VAS, age, sex, BMI, and Kellgren–Lawrence grade. Relative to the 1-week reference, the Group × Time interaction was not significant at 1 month (β = +0.045, *p* = 0.603) or 3 months (β = −0.022, *p* = 0.800), and reached significance only at the 6-month primary endpoint (β = −0.286, 95% CI −0.455 to −0.117; *p* = 0.001), indicating a further widening of the between-group pain difference in favour of IAS + PRF by 6 months. A continuous-time sensitivity analysis was directionally consistent (Group × Week β = −0.013, *p* < 0.001). Kellgren–Lawrence grade independently predicted higher pain (β = 1.017, *p* < 0.001); age, sex, and BMI were not significant. Key coefficients are summarised in Table 5.

### 3.5. Clinical Response Rates and MCID

**Figure 3:**
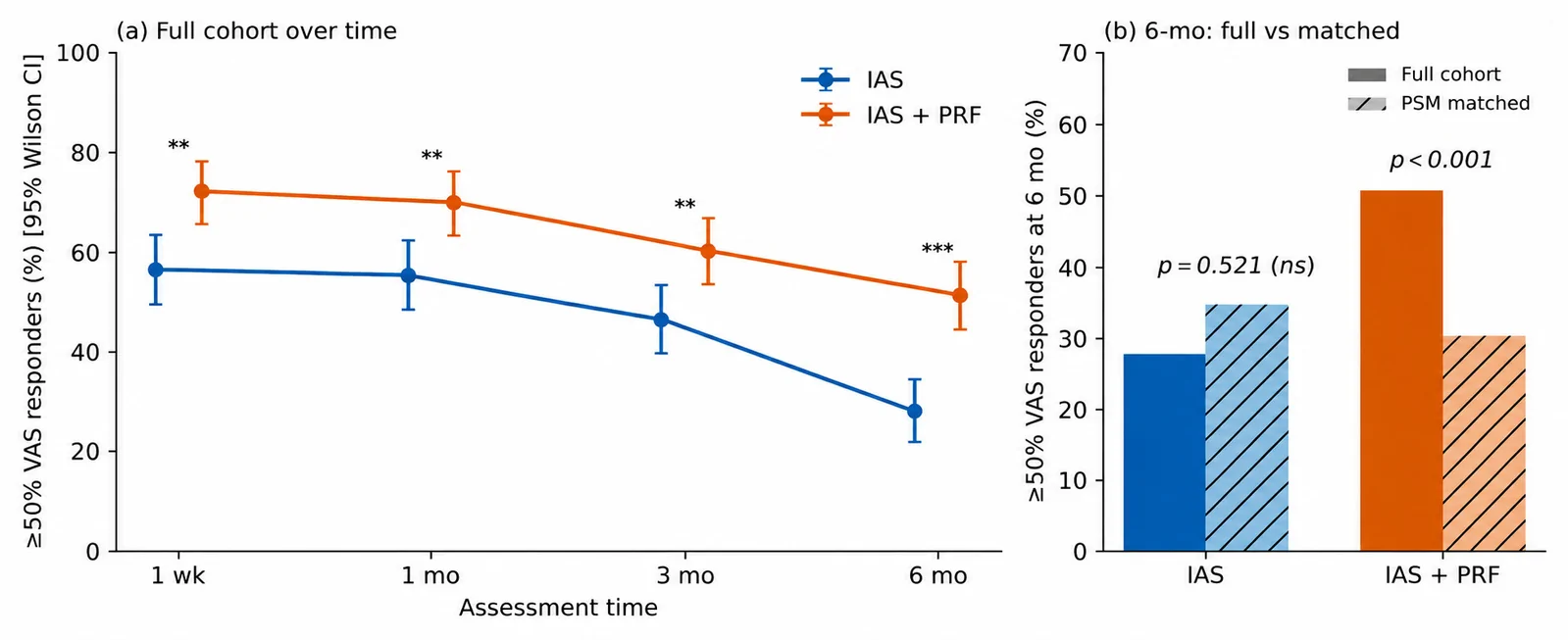
(**a**) ≥50% VAS responder rates over follow-up with 95% Wilson confidence intervals. (**b**) 6-month ≥ 50% responder rates in the full cohort versus the propensity score-matched cohort, showing attenuation of the between-group difference after matching. χ^2^ tests; ns, *p* ≥ 0.05, ** *p* < 0.01, *** *p* < 0.001.

At the primary endpoint (6 months), MCID achievement (VAS reduction ≥ 1.5 points) was higher with IAS + PRF than IAS (80.6% vs. 70.0%; difference +10.6 points, 95% CI 2.0 to 19.1; *p* = 0.021). The more stringent ≥ 50% VAS responder threshold showed significant advantages for IAS + PRF at every time point, most notably at 6 months (51.0% vs. 27.9%; difference +23.1 points, 95% CI 13.4 to 32.2; *p* < 0.001). The lower bound of this CI (+13.4 points) remains clinically substantial under the most conservative interpretation. The ≥ 30% threshold was significant at 6 months (65.8% vs. 44.2%; difference +21.6 points, 95% CI 11.7 to 30.9; *p* < 0.001). Full data are shown in Table 6 and Figure 3.

**Figure 4:**
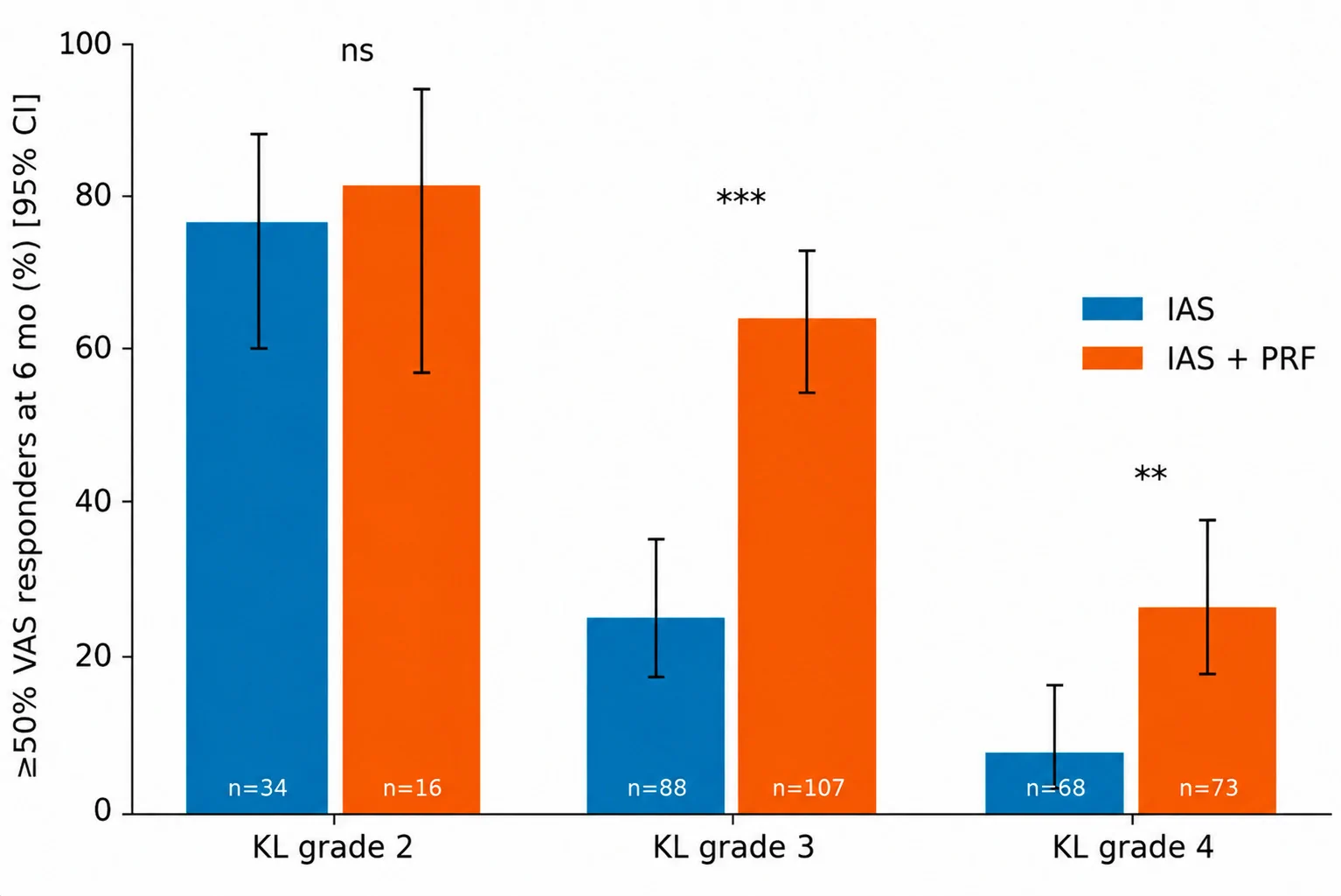
Kellgren–Lawrence-stratified ≥50% VAS responder rates at 6 months, with 95% Wilson confidence intervals. The between-group advantage is concentrated in grade 3–4 disease; grade 2 is inconclusive (IAS + PRF *n* = 16). χ^2^ tests; ns, *p* ≥ 0.05, ** *p* < 0.01, *** *p* < 0.001.

Kellgren–Lawrence stratified analysis (≥50% VAS responders at 6 months). The treatment advantage was most pronounced in Grade 3 (63.6% [54.1–72.1] vs. 25.0% [17.1–35.0]; difference +38.6 points, 95% CI 24.8–50.1; *p* < 0.001) and Grade 4 disease (26.0% [17.3–37.1] vs. 7.4% [3.2–16.1]; difference +18.7 points, 95% CI 6.4–30.5; *p* = 0.006). In Grade 2 patients, both groups had high responder rates (81.2% vs. 76.5%; difference +4.8 points, 95% CI −21.9 to 25.2; *p* = 0.988); given the small sample in this stratum (*n* = 16 in the PRF arm) and the correspondingly wide CI, this result should be interpreted as inconclusive rather than as evidence of therapeutic equivalence (Table 7; Figure 4).

### 3.6. Propensity Score Matching Sensitivity Analysis

To assess robustness under baseline heterogeneity, 1:1 propensity score matching yielded 138 matched pairs (*n* = 276) within the specified calliper. Before matching, baseline WOMAC (SMD = 0.416, 95% CI 0.217–0.626), baseline VAS (SMD = 0.245, 95% CI 0.045–0.447), and Kellgren–Lawrence grade (SMD = 0.165, 95% CI −0.030 to 0.372) exceeded the conventional 0.10 threshold. After matching, the WOMAC imbalance was substantially reduced (SMD = 0.047, 95% CI −0.172 to 0.282) and the bootstrap 95% CI for every post-matching covariate SMD included zero (Table 8), although the point estimates for age (SMD = 0.109) and Kellgren–Lawrence grade (SMD = 0.127) remained marginally above the 0.10 threshold. In the matched cohort, median VAS scores did not differ significantly at any time point (all *p* > 0.35; e.g., 6-month VAS median 5.0 in both groups, Hodges–Lehmann difference 0.00, 95% CI −1.00 to 0.00, *p* = 0.763), and ≥50% VAS responder rates were similar (34.8% vs. 30.4%; *p* = 0.521). Thus, the pain advantage seen in the full cohort was not evident after matching.

Because VAS is measured on a discrete integer scale (0–10), Hodges–Lehmann confidence intervals for the matched-cohort VAS comparisons were narrow or degenerate at several time points; this reflects the resolution limit of an ordinal integer scale and should not be over-interpreted as strong evidence of equivalence. The attenuation of between-group differences in the matched cohort indicates that baseline heterogeneity—particularly in WOMAC and KL grade—partly contributed to the differences seen in the full cohort. These findings do not imply therapeutic equivalence but underscore the limitations of a retrospective non-randomised design, the reduced power of a 138-pair subsample, and the need for confirmation in prospective randomised trials.

### 3.7. Safety

All 386 procedures were performed without intraoperative technical complications. No serious adverse events (joint infection, anaphylaxis, neurological deficit, or systemic corticosteroid toxicity) were recorded during the 6-month follow-up in either group. Transient post-injection pain exacerbation was noted in a small number of patients in both groups and resolved spontaneously within 24–72 h without intervention. No patient required hospitalisation or additional medical treatment attributable to the study procedures.

## 4. Discussion

In the present study, the addition of PRF to intra-articular corticosteroid injection was associated with greater early- and mid-term reductions in pain intensity than corticosteroid injection alone in baseline-adjusted analyses. Both strategies produced early symptomatic improvement, and the linear mixed-effects model showed a significant time-by-treatment interaction at 6 months. However, this adjusted between-group effect was modest (VAS Group × Time β = −0.286 at 6 months), smaller than the pre-specified minimum clinically important difference of 1.5 points. Importantly, propensity score matching was performed as a sensitivity analysis to minimise the influence of the apparent baseline heterogeneity between the treatment cohorts. After propensity score matching, the treatment advantage observed in the primary analysis was no longer evident, and no statistically significant between-group difference in VAS outcomes was observed in the matched cohort. This attenuation of the treatment effect after matching suggests that the apparent benefit observed in the unmatched cohort may have been influenced, at least in part, by baseline differences between the groups. Longitudinal functional (WOMAC) outcomes were not analysed because reliable follow-up WOMAC data were unavailable. Taken together, these findings suggest that PRF may provide an incremental analgesic benefit when used as an adjunct to intra-articular corticosteroid injection in selected patients with knee osteoarthritis. Nevertheless, because this association was attenuated after propensity score matching, the clinical significance of the observed treatment effect should be interpreted with appropriate caution, as baseline differences between the treatment groups may have contributed to the apparent treatment advantage observed in the unmatched cohort. Accordingly, when considering the clinical implications of the present findings, particular attention should be given to the results of the propensity score–matched analysis, which was specifically undertaken to minimise the influence of baseline imbalance. Prospective, adequately powered randomised controlled trials are warranted to confirm these findings.

Our findings are broadly consistent with previous clinical evidence evaluating the addition of PRF to intra-articular corticosteroid injection in patients with knee osteoarthritis. In particular, Erken et al. [34] reported superior pain relief and functional improvement following the addition of PRF, with clinically meaningful benefits persisting at both 4 and 12 weeks. Although differences in study design, patient characteristics, and outcome assessment preclude direct comparison between studies, our baseline-adjusted analyses demonstrated a similar overall pattern of improved pain outcomes with the addition of PRF to intra-articular corticosteroid injection. However, unlike the consistently favourable results reported by Erken et al., the apparent treatment advantage observed in our study was attenuated after propensity score matching, indicating that baseline heterogeneity may have influenced the between-group differences observed in the unmatched cohort. Taken together, these findings suggest that the existing evidence, including the present study, supports the potential role of adding PRF to intra-articular corticosteroid injection in selected patients with knee osteoarthritis, while also underscoring the importance of carefully accounting for baseline imbalance when interpreting comparative treatment effects in observational studies.

Despite the overall consistency between our findings and previous clinical studies, the pattern of functional recovery observed in our cohort warrants further consideration. Rather than demonstrating a clear difference in overall functional improvement between treatment strategies, the principal distinction observed in our cohort was the temporal profile of recovery. The addition of PRF to intra-articular corticosteroid injection was characterised by earlier functional improvement followed by subsequent stabilisation, whereas intra-articular corticosteroid injection alone demonstrated a more gradual pattern of functional recovery throughout follow-up. These observations suggest that reductions in pain intensity may not necessarily be accompanied by proportional improvements in physical function, emphasising that functional recovery in knee osteoarthritis is likely influenced by multiple interacting biological, mechanical, and behavioural factors beyond pain relief alone.

Another notable finding was the independent association between radiographic disease severity and pain outcomes over time. In the adjusted longitudinal analysis, Kellgren–Lawrence grade was an independent predictor of pain, indicating that baseline structural disease burden remained an important determinant of pain response during follow-up. This differs from Erken et al., who reported no significant difference in treatment response between Kellgren–Lawrence grade II and III disease, suggesting that the effects of PRF may be relatively independent of moderate radiographic severity [34]. The discrepancy may reflect differences in design, patient selection, statistical methodology, or baseline disease characteristics, and underscores the importance of considering radiographic severity when interpreting outcomes after intra-articular interventions for knee osteoarthritis.

The findings of Gulec et al. further suggest that procedural factors may influence the clinical response to intra-articular PRF. In their comparison of unipolar and bipolar PRF techniques, both approaches produced significant reductions in pain, but bipolar PRF achieved clinically meaningful pain relief (≥50%) in a higher proportion of patients [35]. Direct comparison with our findings is limited by differences in PRF modality, design, outcome assessment, and statistical methodology; nonetheless, their results are consistent with the possibility that technical aspects of PRF delivery contribute to variability in treatment response. In our cohort, any pain difference associated with the addition of PRF was attenuated after propensity score matching, suggesting that baseline characteristics may have influenced the observed treatment effect. These observations further highlight the need for standardized prospective studies to determine how procedural parameters and patient characteristics influence the response to PRF as an adjunctive treatment.

The comparative study by Hong et al. provides an additional perspective on the role of intra-articular PRF within the spectrum of interventional treatments for knee osteoarthritis. Although they reported comparable pain reduction with genicular nerve radiofrequency thermocoagulation and intra-articular PRF at 3 and 6 months, intra-articular PRF offers pain modulation without irreversible neural destruction, distinguishing it mechanistically from neuroablative procedures [36]. Rather than suggesting superiority over alternative techniques, and recognising that the pain difference associated with adding PRF in our cohort was modest and not robust to matching, these observations at most support a possible role for adding PRF as a minimally invasive adjunctive option to be considered within a comprehensive multimodal management strategy for selected patients.

The studies by Masala et al. further highlight the potential influence of procedural parameters on the clinical response to PRF. Using standardized stimulation settings, they reported sustained pain reduction following single-electrode intra-articular PRF, whereas a subsequent retrospective series employing similar stimulation characteristics but a longer application duration demonstrated clinically meaningful pain relief (≥50%) in 35.5% of patients [37]. Although direct comparisons with our study are limited by differences in PRF protocols, study design, and outcome assessment, these findings complement our results by suggesting that optimization of PRF delivery parameters may represent an additional opportunity to improve clinical outcomes. Future prospective studies should carefully consider both procedural parameters and patient-related factors when evaluating the addition of PRF to intra-articular corticosteroid injection, as a more standardized approach may facilitate more reliable comparisons across studies and contribute to the development of more consistent treatment protocols.

A recent comprehensive review further emphasises that contemporary management of knee osteoarthritis should move beyond isolated intra-articular interventions and instead adopt a whole-joint, multimodal approach reflecting the complex biological and biomechanical nature of the disease [38]. Consistent with this view, knee osteoarthritis is now recognised as a heterogeneous whole-joint disorder involving cartilage, subchondral bone, synovium, periarticular soft tissues, and systemic and biomechanical factors rather than a simple consequence of cartilage wear and tear [39]. Accordingly, no single intra-articular intervention should be regarded as a stand-alone or disease-modifying therapy; rather, intra-articular treatments should be considered adjunctive symptomatic interventions within an individualized multimodal management strategy [40,41,42]. Within this context, the addition of PRF to intra-articular corticosteroid injection should be viewed as a minimally invasive adjunctive option that complements, rather than replaces, established evidence-based conservative management strategies [42,43].

Despite the well-established anti-inflammatory effects of intra-articular corticosteroids, their relatively limited intra-articular residence time means that any pain difference associated with the addition of PRF in the present study is unlikely to be attributable solely to corticosteroid exposure [44,45]; however, given the non-randomized design and the attenuation of the difference after matching, this remains a hypothesis rather than a demonstrated mechanism.

Unlike conventional radiofrequency thermocoagulation, PRF provides neuromodulation without inducing thermal neurodestruction, thereby preserving neural integrity while modulating pain pathways. Because intra-articular lidocaine was administered as part of both treatment strategies, its immediate analgesic effects were likely balanced between groups, making it unlikely that the observed differences in pain trajectory were attributable solely to local anaesthetic exposure. Experimental studies have suggested that PRF may upregulate endogenous opioid precursor expression, suggesting activation of intrinsic analgesic pathways [46]. In parallel, preclinical evidence indicates that pulsed electric fields may influence chondrocyte proliferation and extracellular matrix synthesis under specific experimental conditions [47].

Because the apparent treatment difference was attenuated after propensity score matching and no mechanistic data were collected, the biological basis of any potential PRF effect remains uncertain. Future prospective randomized studies incorporating standardized PRF protocols together with mechanistic and tissue-level assessments are warranted to determine whether PRF exerts a genuine effect in knee osteoarthritis and, if so, to clarify its mechanism and define the patients most likely to benefit.

Future prospective randomized studies are needed to evaluate multimodal treatment strategies that incorporate intra-articular interventions within the broader management of knee osteoarthritis, reflecting the multifactorial nature of the disease [43,48]. Such approaches may combine pharmacological therapy, intra-articular procedures, structured exercise therapy, rehabilitation, weight management, and optimization of other modifiable risk factors. In addition to establishing whether the addition of PRF confers clinical efficacy, these studies may help define which treatment combinations are associated with the greatest benefit in different patient subgroups and facilitate the development of practical, evidence-based treatment algorithms.

This study has several limitations. First, its retrospective, non-randomized, single-center design introduces the potential for selection bias and limits causal inference; notably, the full-cohort between-group pain difference did not persist after propensity score matching, indicating that residual confounding cannot be excluded (including a marginally higher baseline VAS in the IAS + PRF group, which may contribute through regression to the mean) despite longitudinal mixed-effects modelling and propensity score matching. Second, the absence of a sham PRF control group represents a fundamental limitation; without a placebo-controlled arm, the specific neuromodulatory contribution of PRF cannot be distinguished from the effects of the procedure itself, patient expectations, or the intra-articular corticosteroid injection administered in both groups. Third, concomitant use of analgesic or nonsteroidal anti-inflammatory medications during follow-up was not systematically recorded and could not be accounted for; because pain was the primary endpoint, this is a first-order potential confounder, and the pain findings should be interpreted with corresponding caution. Fourth, longitudinal WOMAC outcomes could not be analysed because reliable follow-up functional data were unavailable, so this study is limited to pain outcomes. Fifth, VAS is an ordinal 0–10 integer scale that was modelled as a continuous score in the mixed-effects analysis; although the direction of effect was supported by nonparametric cross-sectional comparisons, this parameterisation is an approximation. Sixth, the six-month follow-up permits evaluation of short- and medium-term outcomes only; studies with follow-up of at least 12–24 months are needed to determine whether any early pain advantage is sustained, attenuated, or reversed over time. Seventh, no formal a priori sample-size calculation was performed, and analyses beyond the pre-specified primary endpoint were exploratory and not corrected for multiple comparisons, so secondary and subgroup findings should be regarded as hypothesis-generating. Finally, although experimental studies support several neuromodulatory and tissue-level mechanisms of PRF, no biological, imaging-based, or histological assessments were performed. Together, these limitations underscore the need for prospective, randomized, sham-controlled trials with standardized protocols, longer follow-up, and mechanistic outcome measures to establish causality and clarify the effects of adding PRF to intra-articular corticosteroid injection in knee osteoarthritis.

## 5. Conclusions

In this retrospective comparative study, adding PRF to intra-articular corticosteroid injection was associated with more favourable early- and mid-term pain outcomes than intra-articular corticosteroid injection alone in the unmatched cohort; however, this apparent treatment advantage was attenuated after propensity score matching. Although these findings suggest that adding PRF to intra-articular corticosteroid injection may represent a useful minimally invasive adjunctive treatment within an individualized multimodal management strategy for selected patients, they should be interpreted cautiously given the retrospective, non-randomized study design, baseline differences between treatment groups, and the potential for residual confounding. Accordingly, future prospective, randomized, sham-controlled studies incorporating standardized treatment protocols, multimodal treatment strategies, and follow-up of at least 12–24 months are warranted to confirm these findings, determine whether the early pain advantage associated with the addition of PRF is sustained, attenuated, or reversed over time, and further clarify the role of adding PRF to intra-articular corticosteroid injection in the contemporary management of knee osteoarthritis.

## Figures and Tables

**Table 1:** Baseline demographic and clinical characteristics.

Characteristic	IAS (*n* = 190)	IAS + PRF (*n* = 196)	*p*
Age, years (mean ± SD)	69.71 ± 10.62	69.76 ± 9.43	0.965
BMI, kg/m^2^ (mean ± SD)	31.62 ± 2.92	31.75 ± 2.70	0.636
Female, *n* (%)	134 (70.5)	138 (70.4)	1.000
Baseline VAS, median (IQR)	8.0 (7.0–8.0)	8.0 (8.0–8.0)	0.040
Baseline WOMAC, median (IQR)	65.0 (45.0–80.0)	50.0 (35.5–70.0)	<0.001

SD, standard deviation; IQR, interquartile range. *t*-test (age, BMI), Mann–Whitney U (VAS, WOMAC), chi-square (sex).

**Table 2:** Kellgren–Lawrence grade distribution.

KL Grade	IAS, *n* (%)	IAS + PRF, *n* (%)	Total, *n* (%)
Grade 2	34 (17.9)	16 (8.2)	50 (13.0)
Grade 3	88 (46.3)	107 (54.6)	195 (50.5)
Grade 4	68 (35.8)	73 (37.2)	141 (36.5)

Between-group distribution: chi-square *p* = 0.015.

**Table 3:** Cross-sectional VAS scores, median (IQR).

Time	IAS (VAS)	IAS + PRF (VAS)	*p* (VAS)
1 wk	4.0 (3.0–5.0)	3.0 (2.0–4.5)	0.002
1 mo	4.0 (3.0–5.0)	3.0 (2.0–5.0)	0.004
3 mo	4.0 (3.0–6.0)	4.0 (2.0–5.0)	0.003
6 mo	6.0 (4.0–7.0)	4.0 (3.0–6.0)	<0.001

Mann–Whitney U test.

**Table 4:** Change from baseline at 6 months (median, IQR).

Metric	IAS	IAS + PRF	*p*
Δ VAS (points)	−2 (−4 to −1)	−4 (−5 to −2)	<0.001
Δ VAS (%)	−28.6 (−50.0 to −13.0)	−50.0 (−62.5 to −25.0)	<0.001

Mann–Whitney U test on patient-level change scores. Negative values denote improvement.

**Table 5:** Key VAS model coefficients (baseline-adjusted linear mixed-effects model).

Model	β	95% CI	*p*
VAS, baseline-adjusted LMM	−0.286	−0.455 to −0.117	0.001
VAS, main effect of group	−0.718	—	<0.001

Negative coefficients favour IAS + PRF. Covariates: baseline score, age, sex, BMI, KL grade.

**Table 6:** MCID and VAS responder rates, % [95% CI].

Metric/Time	IAS % [95% CI]	IAS + PRF % [95% CI]	Diff [95% CI]	*p*
MCID 3 mo	81.1 [74.9–86.0]	88.8 [83.6–92.5]	+7.7 [0.6, 14.9]	0.048
MCID 6 mo	70.0 [63.1–76.1]	80.6 [74.5–85.5]	+10.6 [2.0, 19.1]	0.021
≥30% 6 mo	44.2 [37.3–51.3]	65.8 [58.9–72.1]	+21.6 [11.7, 30.9]	<0.001
≥50% 1 wk	56.8 [49.7–63.7]	71.9 [65.3–77.8]	+15.1 [5.5, 24.3]	0.003
≥50% 1 mo	55.8 [48.7–62.7]	69.9 [63.1–75.9]	+14.1 [4.5, 23.4]	0.006
≥50% 3 mo	46.8 [39.9–53.9]	60.2 [53.2–66.8]	+13.4 [3.4, 23.0]	0.011
≥50% 6 mo	27.9 [22.0–34.7]	51.0 [44.1–57.9]	+23.1 [13.4, 32.2]	<0.001

Wilson score CI (proportions); Newcombe CI (differences); chi-square (*p*). MCID = VAS reduction ≥1.5 points.

**Table 7:** KL-stratified ≥50% VAS responder rates at 6 months, % [95% CI].

KL Grade	IAS % [95% CI] (*n*)	IAS + PRF % [95% CI] (*n*)	Diff	*p*
Grade 2	76.5 [60.0–87.6] (34)	81.2 [57.0–93.4] (16)	+4.8	0.988
Grade 3	25.0 [17.1–35.0] (88)	63.6 [54.1–72.1] (107)	+38.6	<0.001
Grade 4	7.4 [3.2–16.1] (68)	26.0 [17.3–37.1] (73)	+18.7	0.006

Grade 2 result is inconclusive owing to limited sample size (95% CI for difference −21.9 to 25.2).

**Table 8:** Standardised mean differences (SMD) before and after matching.

Covariate	SMD Before [95% CI]	SMD After [95% CI]
Age	0.009 [−0.184, 0.207]	0.109 [−0.115, 0.339]
Sex	0.009 [−0.187, 0.208]	−0.016 [−0.252, 0.224]
BMI	0.046 [−0.147, 0.241]	0.009 [−0.219, 0.232]
KL grade	0.165 [−0.030, 0.372]	0.127 [−0.098, 0.358]
Baseline VAS	0.245 [0.045, 0.447]	0.130 [−0.100, 0.368]
Baseline WOMAC	0.416 [0.217, 0.626]	0.047 [−0.172, 0.282]

Bootstrap 95% CI (2000 iterations). After matching, all CIs include zero.

## Data Availability

The data presented in this study are available on reasonable request from the corresponding author. The data are not publicly available due to privacy and ethical restrictions.

## Supplementary Materials

The following supporting information can be downloaded at: https://www.mdpi.com/article/10.3390/medicina62081492/s1, Table S1: STROBE Statement—checklist of items that should be included in reports of observational studies.
