# Supplementary material for: Intra-Articular Steroid Injection with or Without Pulsed Radiofrequency in Knee Osteoarthritis: A Retrospective Comparative Study
Source: Medicina (Kaunas). 2026 Aug 3;62(8):1492. doi: 10.3390/medicina62081492 (PMC13514880; doi:10.3390/medicina62081492)
Supplement: Supplementary file 1 [file medicina-62-01492-s001.zip › medicina-4319874-supplementary.pdf]

Table S1. STROBE Statement—checklist of items that should be included in reports of observational studies

	Item No.	Recommendation	Page No.	Relevant text from manuscript
**Title and abstract**	1	(*a*) Indicate the study's design with a commonly used term in the title or the abstract	1	Intra-Articular Steroid İnjection with or Without Pulsed Radiofrequency in Knee Osteoarthritis: A Retrospective Comparative Study
		(*b*) Provide in the abstract an informative and balanced summary of what was done and what was found	1	This retrospective study included 386 consecutive patients with knee osteoarthritis who underwent fluoroscopyguided intra-articular corticosteroid injection alone (IAS, n = 190) or intra-articular corticosteroid injection combined with pulsed radiofrequency (IAS + PRF, n = 196).
**Introduction**				

Background/rationale	2	Explain the scientific background and rationale for the investigation being reported	2-3	The Introduction describes knee osteoarthritis as a whole-joint disease, reviews the limitations of current treatment options, explains the biological rationale for pulsed radiofrequency (PRF), and highlights the limited evidence regarding its combination with intra-articular corticosteroid injection.
				corticosteroids, remains insufficient.
Objectives	3	State specific objectives, including any prespecified hypotheses	3	The study aimed to compare pain outcomes after intra-articular corticosteroid injection alone versus corticosteroid injection combined with PRF in patients with knee osteoarthritis refractory to conservative treatment.

**Methods**				
Study design	4	Present key elements of study design early in the paper	3-4	This retrospective comparative study was approved by the Institutional Ethics Committee of our institution (E1-24-736, November 20, 2024). Medical records and follow-up documentation of patients with knee osteoarthritis treated between 1 January 2022 and 31 December 2024 were reviewed.
Setting	5	Describe the setting, locations, and relevant dates, including periods of recruitment, exposure, follow-up, and data collection	5	Medical records and follow-up documentation of patients with knee osteoarthritis who underwent fluoroscopy-guided intra-articular injection between 1 January 2022 and 31 December 2024 were reviewed. VAS and WOMAC scores were recorded at baseline, and VAS was assessed at 1 week, 1 month, 3 months, and 6 months.

Participants	6	(*a*) *Cohort study*—Give the eligibility criteria, and the sources and methods of selection of participants. Describe methods of follow-up<br>*Case-control study*—Give the eligibility criteria, and the sources and methods of case ascertainment and control selection. Give the rationale for the choice of cases and controls *Cross-sectional study*—Give the eligibility criteria, and the sources and methods of selection of participants	5-6	A total of 386 consecutive eligible patients with clinically and radiologically confirmed knee osteoarthritis who underwent fluoroscopy-guided IAS or IAS + PRF between 1 January 2022 and 31 December 2024 were included. Eligibility criteria were predefined, and VAS outcomes were assessed at baseline and at 1 week, 1 month, 3 months, and 6 months..
		(*b*) *Cohort study*—For matched studies, give matching criteria and number of exposed and unexposed<br>*Case-control study*—For matched studies, give matching criteria and the number of controls per case	N/A	Not applicable (not a matched study).
Variables	7	Clearly define all outcomes, exposures, predictors, potential confounders, and effect modifiers. Give diagnostic criteria, if applicable	5-8	Pain intensity was assessed using the VAS at baseline and at 1 week, 1 month, 3 months, and 6 months. Baseline functional status was assessed using the WOMAC index. The treatment group (IAS vs IAS + PRF) was the primary exposure, and age, sex, BMI, Kellgren–Lawrence grade, baseline VAS, and baseline WOMAC were included as covariates in the adjusted analyses.

Data sources/ measurement	8*	For each variable of interest, give sources of data and details of methods of assessment (measurement). Describe comparability of assessment methods if there is more than one group	6-8	Medical records and follow-up documentation were reviewed. Pain intensity was assessed using the 10-point Visual Analog Scale (VAS) at baseline and at 1 week, 1 month, 3 months, and 6 months. Baseline functional status was assessed using the Western Ontario and McMaster Universities Osteoarthritis Index (WOMAC). The same assessment methods were applied in both treatment groups.
Bias	9	Describe any efforts to address potential sources of bias	5-14	Potential sources of bias were addressed by using standardized treatment procedures, baseline-adjusted linear mixed-effects models, and propensity score matching. Age, sex, BMI, Kellgren–Lawrence grade, baseline VAS, and baseline WOMAC were included as covariates. The limitations of the retrospective, non-randomized, single-center design and the possibility of residual confounding were acknowledged in the Discussion.

Study size	10	Explain how the study size was arrived at	5-6	All consecutive eligible patients who met the predefined inclusion criteria during the study period were included. A total of 386 patients were analyzed and allocated to the IAS group (n = 190) or the IAS + PRF group (n = 196). No formal sample size calculation was performed.s

Continued on next page

Quantitative variables	11	Explain how quantitative variables were handled in the analyses. If applicable, describe which groupings were chosen and why	6-9	VAS scores were analyzed as continuous variables at baseline and at 1 week, 1 month, 3 months, and 6 months. Changes from baseline were also calculated. Baseline WOMAC scores were analyzed as continuous variables and included as a covariate in the adjusted analyses. In the linear mixed-effects models, time was treated as a categorical repeated factor, and age, sex, BMI, Kellgren–Lawrence grade, baseline VAS, and baseline WOMAC were included as covariates.

Statistical methods	12	(*a*) Describe all statistical methods, including those used to control for confounding	5-6	Baseline characteristics were compared using the independent-samples t-test, Mann–Whitney U test, or Pearson's chi-square test, as appropriate. Cross-sectional between-group comparisons at each follow-up time point were performed using the Mann–Whitney U test. Longitudinal VAS outcomes were analyzed using baseline-adjusted linear mixed-effects models including treatment group, time, and Group × Time interaction as fixed effects, with age, sex, BMI, Kellgren–Lawrence grade, baseline VAS, and baseline WOMAC as covariates and subject-specific random intercepts. Propensity score matching was performed as a sensitivity analysis.
		(*b*) Describe any methods used to examine subgroups and interactions	6–7, 11–12	The Group × Time interaction was evaluated in the baseline-adjusted linear mixed-effects models. Prespecified subgroup analyses were performed according to Kellgren–Lawrence grade (grades 2, 3, and 4). Propensity score-matched analyses were also performed as sensitivity analyses.

	(*c*) Explain how missing data were addressed	5-6	Patients without 6-month follow-up data were excluded before analysis. No statistical imputation was performed. Linear mixed-effects models were used for the repeated-measures analyses of the available data.
	(*d*) *Cohort study*—If applicable, explain how loss to follow-up was addressed<br>*Case-control study*—If applicable, explain how matching of cases and controls was addressed *Cross-sectional study*—If applicable, describe analytical methods taking account of sampling strategy	5-6	Patients without 6-month follow-up data (n = 16) were excluded during the eligibility assessment before the final study cohort was established. Follow-up outcomes were analyzed only for patients with complete follow-up data.
	(*e*) Describe any sensitivity analyses	6–7, 11–12	Sensitivity analyses included propensity score matching and baseline-adjusted analyses to evaluate the robustness of the primary findings. The consistency of the treatment effect was also examined across Kellgren–Lawrence grade subgroups.
**Results**			

Participants	13*	(a) Report numbers of individuals at each stage of study—eg numbers potentially eligible, examined for eligibility, confirmed eligible, included in the study, completing follow-up, and analysed	4	A total of 418 patients were assessed for eligibility. Thirty-two patients were excluded (16 with no 6-month follow-up, 2 with Kellgren–Lawrence grade I, 11 who received hyaluronic acid injection within the previous 6 months, and 3 who received platelet-rich plasma injection within the previous 6 months). The final study cohort consisted of 386 patients, including 190 in the IAS group and 196 in the IAS + PRF group (Figure 1).
		(b) Give reasons for non-participation at each stage	5	Not applicable. This was a retrospective cohort study based on consecutive eligible patients identified from routine clinical records, and no staged prospective recruitment or participant flow was reported.
		(c) Consider use of a flow diagram	4	A flow diagram was provided.

Descriptive data	14*	(a) Give characteristics of study participants (eg demographic, clinical, social) and information on exposures and potential confounders	6-7	Baseline demographic and clinical characteristics of the study population are presented in Table 1. The IAS and IAS + PRF groups were comparable in terms of age and BMI, whereas baseline VAS scores, baseline WOMAC scores, and KL grade distribution differed significantly between groups in Table 2.
		(b) Indicate number of participants with missing data for each variable of interest.	N/A	The number of participants with missing data for each variable of interest was not reported separately. Analyses were performed using the available repeated-measures data, and linear mixed models were used to accommodate potential missing observations.
		(c) *Cohort study*—Summarise follow-up time (eg, average and total amount)	5	Clinical outcomes were assessed at baseline and at 1 week, 1 month, 3 months, and 6 months. Thus, the planned follow-up period for the cohort extended to 6 months. This information is stated in outcome measures in Methods.

Outcome data	15*	*Cohort study*—Report numbers of outcome events or summary measures over time	7-12	Median VAS scores (IQR) at baseline and at 1 week, 1 month, 3 months, and 6 months are reported in Tables 1–3. Absolute and percentage changes from baseline are presented in Table 4. Baseline-adjusted linear mixed-effects model estimates are presented in Table 5, propensity score-matched analyses in Table 6, and Kellgren–Lawrence-stratified responder outcomes in Table 7 and Figures 3–4.
		Case-control study—Report numbers in each exposure category, or summary measures of exposure		
		Cross-sectional study—Report numbers of outcome events or summary measures		

Main results	16	(*a*) Give unadjusted estimates and, if applicable, confounder-adjusted estimates and their precision (eg, 95% confidence interval). Make clear which confounders were adjusted for and why they were included	7-11	Unadjusted VAS medians and between-group comparisons are reported in Table 3, with absolute and percentage changes from baseline in Table 4. Confounder-adjusted longitudinal analyses were performed using baseline-adjusted linear mixed-effects models including treatment group, time, Group × Time interaction, baseline VAS, age, sex, BMI, and Kellgren–Lawrence grade. Treatment effects are reported with β coefficients, 95% confidence intervals, and p values (Table 5). Propensity score-matched estimates and confidence intervals are additionally presented in Table 6.
		(*b*) Report category boundaries when continuous variables were categorized	N/A	Not applicable. Continuous outcomes were analyzed as continuous variables, and no continuous exposure variables were categorized for the primary analyses.

	(*c*) If relevant, consider translating estimates of relative risk into absolute risk for a meaningful time period	N/A	Not applicable. This study evaluated continuous longitudinal outcomes (VAS and WOMAC) rather than relative risk measures.

Continued on next page

Other analyses	17	Report other analyses done—eg analyses of subgroups and interactions, and sensitivity analyses	9-12	Additional analyses included propensity score-matched comparisons and Kellgren–Lawrence grade-stratified responder analyses. Adjusted longitudinal analyses were performed using baseline-adjusted linear mixed-effects models. Sensitivity analyses included additional baseline-adjusted regression models for the 6-month outcomes to assess the robustness of the findings. No formal subgroup analyses were performed.
**Discussion**				
Key results	18	Summarise key results with reference to study objectives	12-13	In line with the study objective, the addition of intra-articular pulsed radiofrequency to corticosteroid injection was associated with greater and more sustained pain reduction than corticosteroid injection alone in the unmatched analyses. After adjustment for baseline imbalances and propensity score matching, the magnitude of the treatment effect

				was attenuated, although the overall direction of benefit remained consistent, supporting cautious interpretation of the findings in this retrospective observational study.
Limitations	19	Discuss limitations of the study, taking into account sources of potential bias or imprecision. Discuss both direction and magnitude of any potential bias	14	This study has several limitations, including its retrospective, non-randomized, single-center design, which limits causal inference and generalizability. Residual confounding cannot be excluded despite baseline-adjusted and propensity score-matched analyses. The 6-month follow-up does not permit assessment of long-term durability, and the absence of a sham-controlled or placebo group limits attribution of the observed effects specifically to intra-articular pulsed radiofrequency.

Interpretation	20	Give a cautious overall interpretation of results considering objectives, limitations, multiplicity of analyses, results from similar studies, and other relevant evidence	13	The findings are most applicable to patients with symptomatic knee osteoarthritis treated in tertiary interventional pain medicine settings who are candidates for fluoroscopy-guided intra-articular procedures. Generalisability is limited by the retrospective, single-center design, local treatment practices, and the non-randomized treatment allocation.
Generalisability	21	Discuss the generalisability (external validity) of the study results	13	These findings are most applicable to patients with symptomatic knee osteoarthritis treated in interventional pain practice who are candidates for fluoroscopy-guided intra-articular procedures. External validity is limited by the retrospective single-center design and local treatment practices.
**Other information**				
Funding	22	Give the source of funding and the role of the funders for the present study and, if applicable, for the originalstudy on which the present article is based	14	This research received no external funding.

*Give information separately for cases and controls in case-control studies and, if applicable, for exposed and unexposed groups in cohort and cross-sectional studies.

Note: An Explanation and Elaboration article discusses each checklist item and gives methodological background and published examples of transparent reporting. The STROBE checklist is best used in conjunction with this article (freely available on the Web sites of PLoS Medicine at http://www.plosmedicine.org/, Annals of Internal Medicine at http://www.annals.org/, and Epidemiology at http://www.epidem.com/). Information on the STROBE Initiative is available at www.strobe-statement.org.
